# Hardness Distribution of Al2050 Parts Fabricated Using Additive Friction Stir Deposition

**DOI:** 10.3390/ma16031278

**Published:** 2023-02-02

**Authors:** Hamed Ghadimi, Huan Ding, Selami Emanet, Mojtaba Talachian, Chase Cox, Michael Eller, Shengmin Guo

**Affiliations:** 1 Department of Mechanical & Industrial Engineering, Louisiana State University, Baton Rouge, LA 70803, USA; 2 MELD Manufacturing Corporation, 200 Technology Dr, Christiansburg, VA 24073, USA; 3 Lockheed Martin Space-Michoud, New Orleans, LA 70129, USA

**Keywords:** additive manufacturing, additive friction stir deposition, Al2050, microstructure, microhardness

## Abstract

The solid-state additive friction stir deposition (AFSD) process is a layer-by-layer metal 3D-printing technology. In this study, AFSD is used to fabricate Al–Cu–Li 2050 alloy parts. The hardness values for various regions of the as-deposited built parts are measured, and the results are contrasted with those of the feedstock material. The as-fabricated Al2050 parts are found to have a unique hardness distribution due to the location-specific variations in the processing temperature profile. The XRD results indicate the presence of the secondary phases in the deposited parts, and EDS mapping confirms the formation of detectable alloying particles in the as-deposited Al2050 matrix. The AFSD thermal–mechanical process causes the unique hardness distribution and the reduced microhardness level in the AFSD components, in contrast to those of the feedstock material.

## 1. Introduction

Additive friction stir deposition (AFSD) is a layer-by-layer thermomechanical solid-state additive manufacturing (AM) process [1,2,3] that stirs the plastically deformed and softened feedstock metal onto the lower layer(s) to form a three-dimensional part [4,5,6,7,8,9,10]. Known as a fast and scalable thermomechanical processing method, AFSD utilizes mechanically induced plastic deformation and material flow—caused by the heat generated in the friction process—to deposit the feedstock material. The friction between the pushed rotating feedstock rod, deposition tool, and the fixed substrate (build surface [11]) is the main fusion source in AFSD [9,10].

**Figure 1:**
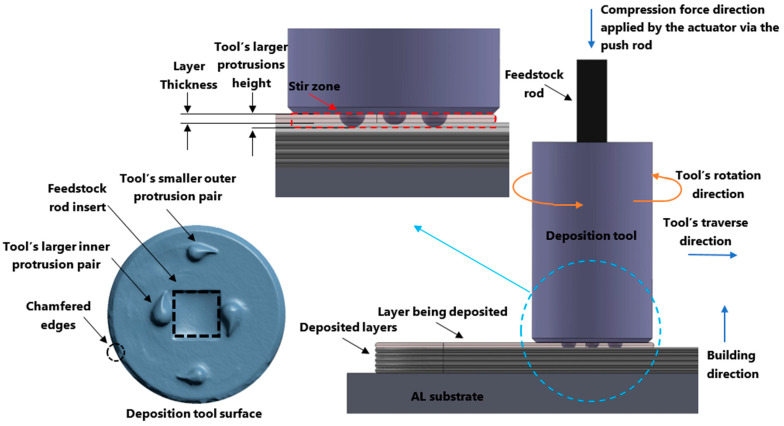
AFSD deposition mechanism and the deposition tool design.

The distinct advantages of AFSD are its high deposition rate, scalability [12], open-air printing, and extensive applicability (e.g., recycling of chips [13] and repairs [14]). Fully dense—with no visible porosity—near-net-shape parts can be fabricated in the single-step AFSD process [12,15]. If necessary, the as-built parts can go through the post-processing stages to remove the flash regions (See Figure 1) or to receive further heat treatment. As the AFSD technique does not induce the melting of the feedstock material [9,16,17], AFSD components do not experience the problems brought on by rapid melting and solidification commonly found in laser powder bed fusion-based AM (e.g., porosity and hot cracking) [5].

In AFSD, refined/equiaxed grains are developed during dynamic recrystallization [5,7,17]. Investigating the microstructure of the AFSD-manufactured Al6061 parts, Zeng et al. concluded that the as-deposited AFSD parts have an equiaxed microstructure that can be further enhanced by heat treatment. The grain sizes in the as-deposited AFSD pieces are much smaller than those in the feedstock rod [18]. Williams et al. [4] investigated how the AFSD processing parameters affect the microstructure and mechanical properties of the magnesium alloy WE43 component. According to their findings, the AFSD component had a refined, uniform microstructure and the average grain size was considerably smaller than that of the feedstock. With many unexplored aspects, friction-based AFSD is a relatively new large-scale AM manufacturing technique [5,7,9,12,15,19].

Friction stir processing (FSP) is a solid-state joining technique that utilizes a rotating non-consumable tool to generate heat and plastic deformation in the material being processed, without reaching melting temperatures, thus, allowing the bonding of two materials together in a solid-state fashion [20]. FSP is an extension of the friction stir welding (FSW) technique [20]. Both AFSD and FSP use a rotating tool to generate heat and rely on the plastic deformation of the material to create a bond between the materials, performed at temperatures below melting. A number of studies were conducted on Al2050 alloy processed by FSW, with the goal of understanding how these processes affect the microstructure and the mechanical properties of the alloy [21,22,23,24].

In transportation and aerospace industries, high-strength aluminum 2XXX series alloys are commonly used [25,26]. These alloys stand out for their remarkable strengths at cryogenic and elevated temperatures, as well as their creep resistance at high temperatures [27]. Al2050 is a wrought aluminum alloy with copper as the major alloying element and is strengthened by solution heat treatment [27,28]. This Al–Cu–Li–Mg–Zr alloy was developed by modifying alloy 2098 and has high tensile strength, good fracture toughness, and strong resistance to stress corrosion cracking [27]. This paper reports the microstructure and hardness of the Al2050 block fabricated by ASFD. To the best of the authors’ knowledge, Al2050 alloy AFSD processing and also microstructure and mechanical properties of the AFSD fabricated parts have not been investigated.

## 2. Additive Friction Stir Deposition of Al2050 Alloy

### 2.1. How an AFSD Machine Functions

An AFSD machine (L3 model, made by MELD Manufacturing Corporation [29]) was used to manufacture the Al2050 specimens with layer thicknesses of 1.5 mm (0.06 in). During the AFSD process, the deposition tool first starts spinning with the feedstock rod within it. Then, the tool is lowered to the working position, which controls the thickness of the deposition layer. Heat is produced as the tool’s teardrop-shaped features of protrusions (~2 mm in height) come into contact with the substrate. Then the push-rod forces the feedstock rod down through the spindle to rub against the substrate, producing further heat to prepare the material for plastic deformation. At the working temperature, feedstock rod yields and is extruded out of the deposition tool. The plastically deformed material flows beneath the spinning tool, filling the space between the tool’s lower face and the substrate (or the previously deposited layer). The protrusions on the tool surface help stir the depositing softened material. While the material flows at a certain rate, the tool traverses along the specified path depositing the material and forming a new layer. Tool spindle speed, tool traverse speed, and material feed rate are the three main deposition parameters that must be configured for a successful disposition (See Figure 1).

Additive friction stir deposition is a thermomechanical processing method as it combines the mechanical process of compression with heat generated in the friction process to deposit the material [30]. The AFSD tool deposits the plastically deformed material at an elevated temperature on top of the prior layer to print the subsequent layer. The thickness of the deposition layer is determined by the distance between the lower face of the tool and the substrate (or the previously deposited layer). The depositing layer may be mixed into the lower layer that has already been deposited (build surface), depending on the setting of the new deposition layer thickness. For this study, the layer thickness is smaller than the protrusion height on the tool surface, so while the new layer is being deposited, a portion of the previous layer is re-stirred. Additionally, by adding a layer on top of an already applied layer, the existing layer is heated up. The microstructure of the impacted region is affected by these reheating and re-stirring practices. Reheating and re-stirring are applied to the entire AFSD parts except for the final top deposited layer.

### 2.2. Feedstock Material Properties

Solution heat-treated Al2050-T84 plates (4 in × 24 in × 0.625 in-thickness) are used for this study. The material composition of the used Al2050 is Al-3.6Cu–1.0Li–0.40Mg–0.35Mn–0.40Ag–0.11Zr–0.25Zn (wt.%) [31,32,33]. The maximum and the minimum for the Al2050 registered chemistry [26] are available in the literature for reference and study. The physical properties of this material are listed in Table 1.

Copper is the major alloying element for the wrought 2050 aluminum alloy. The third-generation A–Li–X alloys [32,33] of 2050 are heat treated to produce stable tempers. The feedstock used for this study is T84 heat-treated [34]. For T84 heat treatment, first, the Al2050 undergoes solution heat treatment at 525 ± 5 °C followed by a water quenching. Then, the alloy goes through a ~4% compressive cold work, and finally, artificial aging at 155 ± 5 °C for 18 h is carried out [34]. The Al2050-T84 has a fine coherent microstructure [35]. The constituents dissolve in the solution during the solution heat treatment. The constituents then remain in the solution while the alloy cools down in the quenching process, serving as the basis for precipitation hardening later during the artificial aging process [36]. Precipitates affect mechanical properties tremendously [28]. The most important precipitate involved in the strengthening of 2050 alloy (Li < 1.4–1.5 wt.%) is the T_1_ (Al_2_CuLi) phase with plate-like morphology [26,33]. Additional contributions in strengthening through precipitation come from $\overset{´}{\delta }$ (Al_3_Li) and $\overset{´}{\theta }$ (Al_2_Cu) phases. Their contribution is less significant than the T_1_ phase and it depends on the lithium content in the alloy. The shearable $\overset{´}{\delta }$ (Al_3_Li) phase contribute to strengthening in alloys with Li > 1.4–1.5 wt.% [26,33,35]. For the lower lithium content (<0.6 wt.%) non-sharable $\overset{´}{\theta }$ (Al_2_Cu) [26,33] phase is present. Al_3_Zr ($\overset{´}{\beta }$) Zr-based dispersoid particles are also present in Al2050 matrix [26]. They develop as a result of the cast ingots’ homogenization treatment. The $\overset{´}{\beta }$ phase (Al_3_Zr) resists recrystallization and provides sites for the nucleation of the $\overset{´}{\delta }$ (Al_3_Li) phase, which causes better mechanical properties through facilitating homogenization of slip [33].

### 2.3. AFSD Parts Sample Preparation

Wrought Al2050-T84 feedstock rods with a square cross-section of 9.5 mm × 9.5 mm (3/8 in × 3/8 in) and a length of 500 mm (20 in) were prepared from large plates using water jet/CNC. Before placing the rods in the AFSD machine, a graphite coating was sprayed on their surface, enabling easier rod movement and preventing the rod from getting stuck in the deposition tool. After setting up the machine and executing the associated G-code, the part was deposited. The deposition parameters, namely, the tool’s rotation speed, the feedstock material feed rate, and the tool’s traverse speed, were held constant to deposit the Al2050 part with a layer thickness of 1.5 mm (0.06 in). The deposited part is composed of thirty layers in the building direction. The part is 228 mm (9 in) in length and the width of the part is 38.1 mm (1.5 in) without considering the flash region, which is equal to the deposition tool’s outside diameter. The part is deposited on a 102 mm × 304 mm (4 in × 12 in), 13 mm (0.5 in) thickness, aluminum alloy substrate plate.

**Figure 2:**
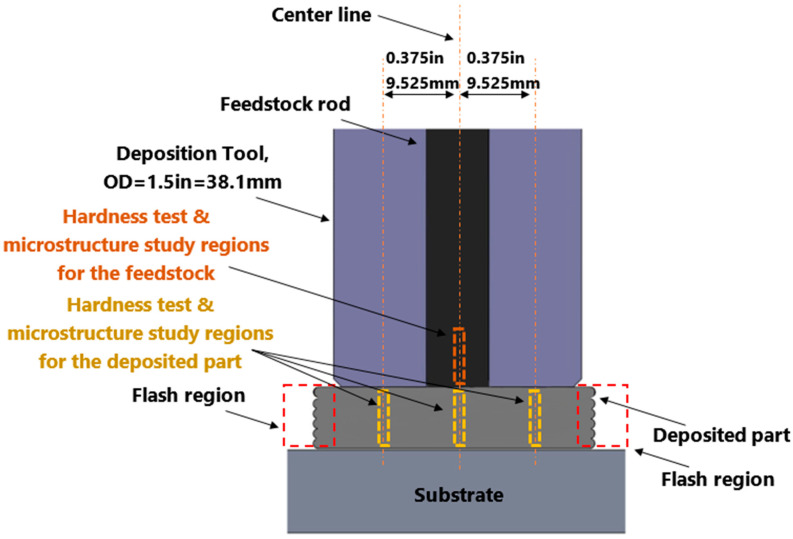
Schematic of the cross-section of the deposited Al2050 part and the feedstock rod, showing the regions chosen for the hardness test and studying the microstructure.

The as-built part was cut using electrical discharge machining (EDM) into 3 mm thickness slices to study the microstructure and mechanical properties. The remaining unprocessed feedstock rod was also sliced to serve as a comparison. Several locations throughout the length of the remaining feedstock rod’s cross section were investigated. The testing locations are shown in Figure 2.

## 3. Results

### 3.1. Layer-by-Layer Arrangement

**Figure 3:**
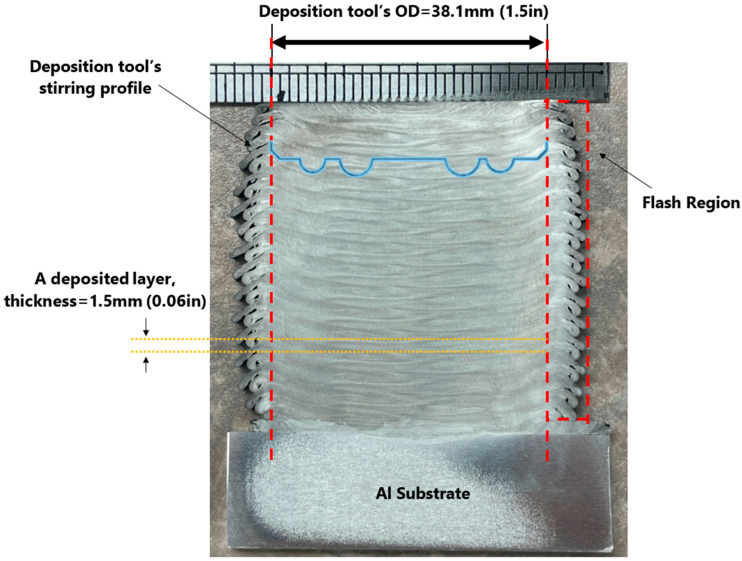
Polished and etched cross-section of the studied Al2050 as-deposited AFSD part showing the 3D layered structures.

The AFSD is a layer-by-layer fabrication method. Using the rotating deposition tool, a new layer is deposited on top of an existing layer. The stirring and heat-generating processes are facilitated by the protrusion features on the deposition tool’s lower face (See Figure 1). In the present investigation, the height of the largest set of protrusions is more than the thickness of the deposited layer. For this reason, a portion of the previous layer is re-stirred while the new layer is deposited. The cross-section of the layers in AFSD-made parts is different from typical layer-by-layer additive manufactured builds due to this re-stirring feature. The layers’ boundaries in the structure of an AFSD part are generally curved and 3D in nature. The stirring and re-stirring mechanism of the AFSD distorts the boundaries of the layer. Figure 3 shows the layered structure for an AFSD part.

There is no reheating or re-stirring for the final top deposited layer. Therefore, the effect of these reheating and re-stirring on the microstructure may be examined by comparing the microstructure of the beneath layers with the top layer along the building direction. The following sections present the findings of Vickers hardness distribution.

### 3.2. Vickers Hardness Distribution

**Figure 4:**
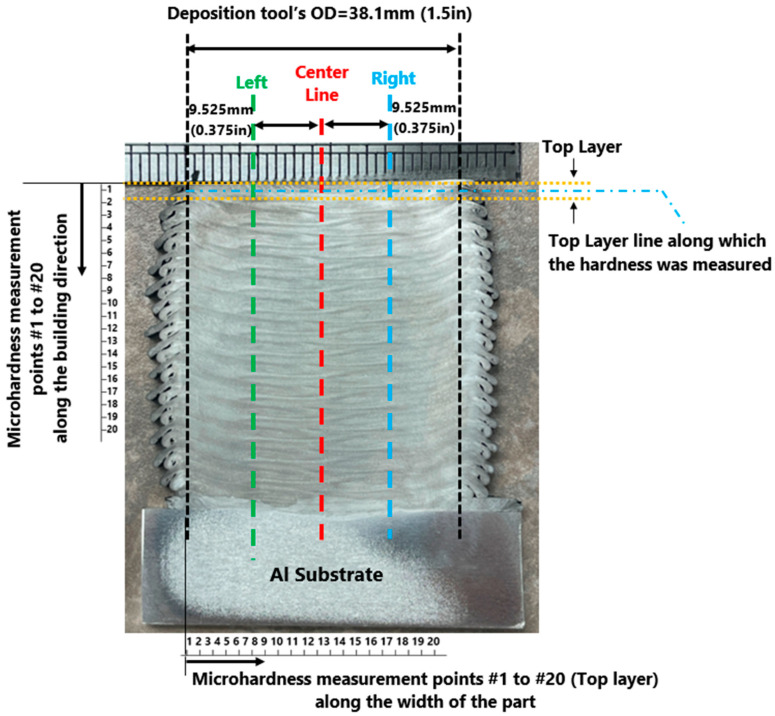
Microhardness measurement points along the building direction for the inner layers and along the as-deposited parts’ cross-section’s width for the top layer.

**Figure 5:**
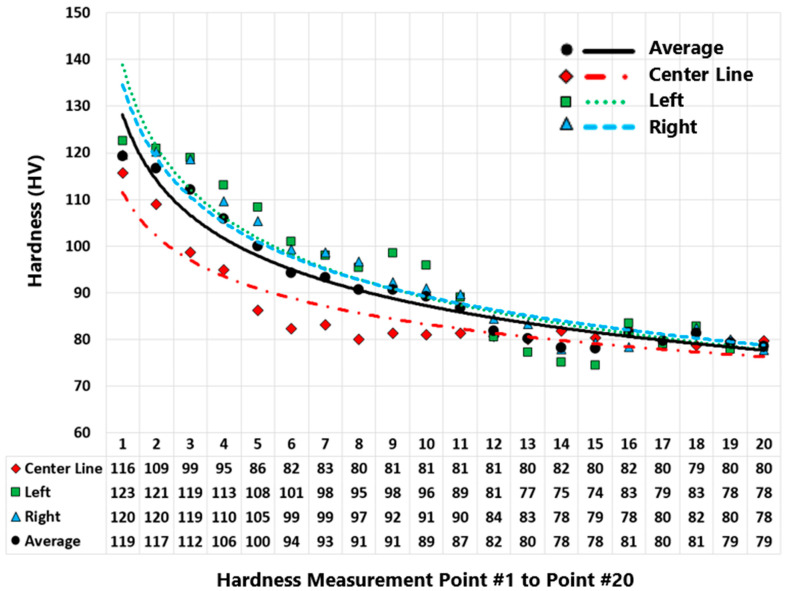
Microhardness results of the as-deposited Al2050 part (cross-section). Measurements along three lines, namely, center, left, and right lines (Figure 4).

**Figure 6:**
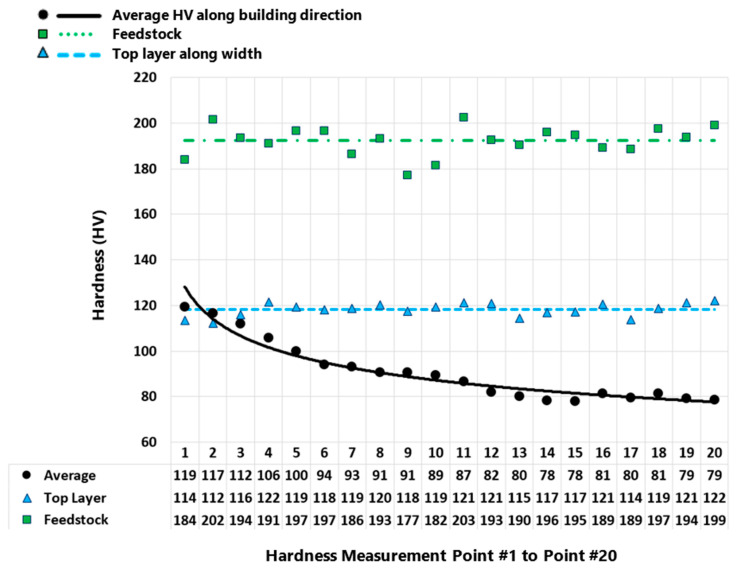
Microhardness results for the as-deposited Al2050 part and the feedstock.

Vickers microhardness test was carried out on the feedstock rod and the as-deposited AFSD part. Figure 4 displays the regions where the microhardness was measured for the as-deposited part. Figure 5 presents the measured values for the as-deposited part. Figure 6 compares the microhardness test results for the feedstock and the as-deposited part. For the Al2050-T84 feedstock, the average of the microhardness values is 192.32 ± 6.52 HV. However, after the AFSD process, the microhardness decreased significantly.

The microhardness measurements were taken from the as-deposited part’s cross-section along the three vertical lines running in the building direction, as shown in Figure 4, starting from the top of the part downward (point #1 is at the top layer and points are ~2 mm apart). In addition, the microhardness test was carried out for the top layer separately (along a horizontal line that is located <3 mm from the top). According to this study’s findings, the measured microhardness values on the part’s cross-section decrease in a nonlinear fashion along the building direction from top to bottom (See Figure 5). The thermal history of the various locations explains the reduction in the hardness through the height of the as-deposited piece. As the top layers are deposited, the lower existing layers go through heating and cooling cycles. As additional layers are printed on top, the bottom layer experiences more of these cycles.

As explained earlier, the top layer may have different properties. The result of this study shows that the mechanical properties of the part in the top layer area are significantly different from the rest of the part (See Figure 6). The top layer (above <1 mm line) area of the deposited part has the highest microhardness values. As discussed earlier, the top layer of the deposited part does not experience the re-stirring and reheating feature of the AFSD process. This may explain the significant difference between the mechanical properties of this region and the rest of the deposited part. Figure 6 displays the feedstock material’s hardness test results in comparison to the top layer and the interior of the deposited part. The average of the microhardness values for the top layer is about 118.16 ± 2.90 HV, which is significantly smaller than the one for the feedstock (192.32 ± 6.52 HV).

## 4. Discussions

This section discusses how the processing temperature history would lead to the unique hardness distribution in the as-fabricated AFSD Al2050 parts.

### 4.1. Phases of Al2050 as a Function of Temperature

**Figure 7:**
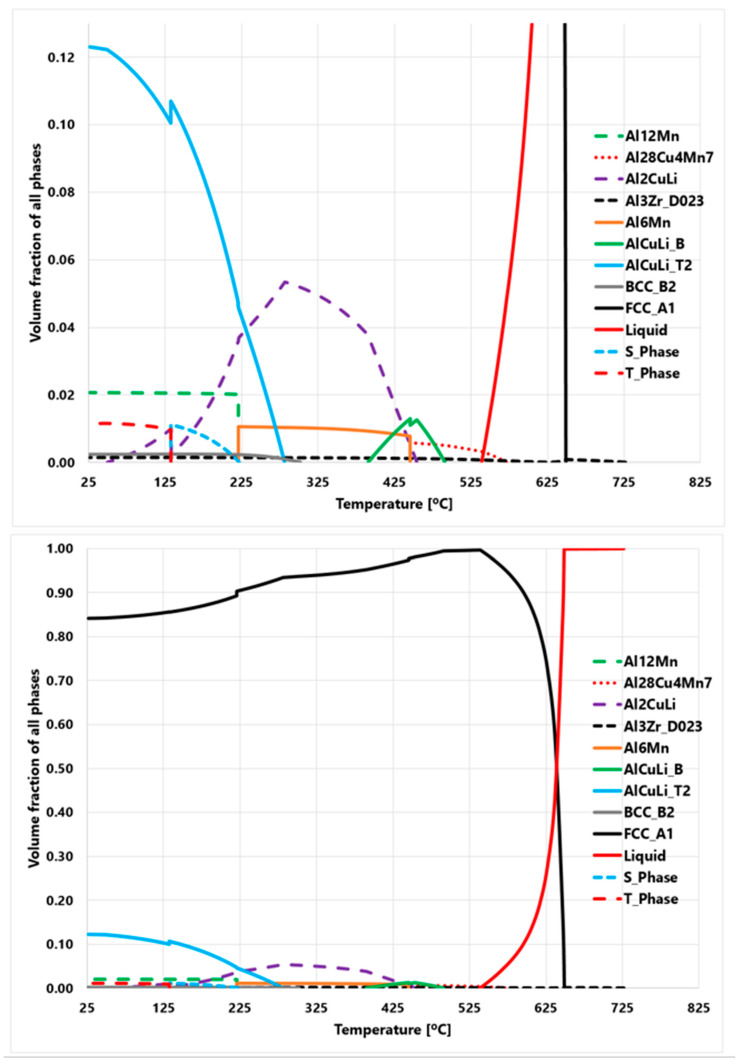
Thermo-Calc prediction results of the Al2050 alloy demonstrating the amounts of phases as a function of temperature.

Figure 7 demonstrates the amount of each phase as a function of temperature for the Al2050 alloy in the equilibrium state. According to the presented Thermal-Calc calculation results, the FCC-Al is the main phase of the Al2050, which is about 85% at room temperature. Also, the T-phase (Al_20_Cu_2_Mn_3_), AlCuLi, BCC, Al_12_Mn, and Al_3_Zr phases are the secondary phases at room temperature. T-phase is a precipitate phase [34].

### 4.2. Deposition Process Temperature History

**Figure 8:**
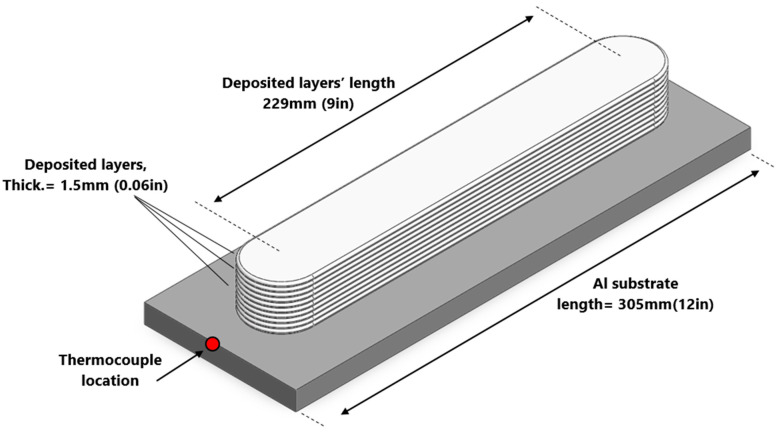
Deposited block geometry and the thermocouple placement to read the temperature during the deposition.

The samples studied in this paper were cut from an as-deposited Al2050 block. The block was printed on a substrate made of aluminum alloy that was 12.7 mm (0.5 in) thick, as shown in Figure 8. Thirty layers were deposited, each layer being 1.5 mm thick (0.06 in). Each layer is deposited along a 229 mm (9 in) long straight line. A thermocouple was installed at the substrate near one end where the deposition starts. The thermocouple readings indicate the temperature at that exact location.

**Figure 9:**
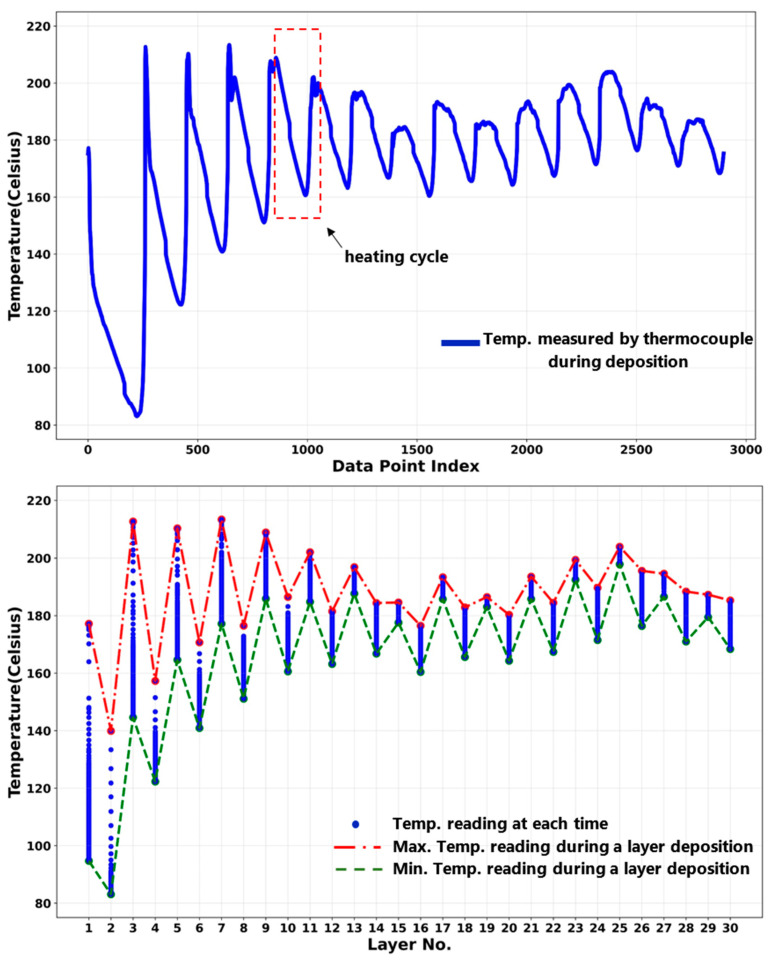
Temperature variation in the substrate during the deposition: (**top**) temperature history as a function of time; (**bottom**) temperature variation as a function of deposited layers.

Figure 9 shows the temperature variation for the location in the substrate where the thermocouple is placed. During the AFSD process, the region just beneath the deposition tool has the maximum temperature. When printing the layers, the deposition tool traverses, causing this high-temperature region to move. The temperature of a location in a beneath layer increases as the tool approaches its position and decreases as the high-temperature region moves away from that location. Adding new layers at the top causes re-heating cycles to occur over the layers beneath. The heating/re-heating cycles’ amplitude decreases when the high-temperature source moves away from a particular spot at a beneath layer. However, adding additional materials to the component (as the part deposition proceeds) implies that the high-temperature zone will remain there for a longer period of time, raising the temperature of the part as a whole (See Figure 9). As Figure 9 suggests, while the layers are being deposited, the mean temperature measured at the thermocouple reading point rises.

Figure 9 (top) shows the heating/re-heating cycles and the gradual increase in the mean temperature of the reading point as the deposition proceeds. Figure 9 (bottom) displays the maximums of temperatures that were recorded during the deposition of each layer. This temperature fluctuates throughout the deposition process, peaking at around the seventh layer, after which it lowers, then rises once again. The utilized AFSD machine has a manual feeding mechanism and each feedstock rod was used to deposit two consecutive layers. That explains why there is a variation in the maximum temperature, since it takes time to change the feedstock rod and the part’s temperature decreases in the meantime. On the other hand, during the deposition, heat continuously dissipates from the surfaces of the components and the substrate. As a result, the maximum temperature peaks rise initially before reaching the plateau at around the seventh layer.

Figure 9 displays the thermocouple reading in the substrate taken during the deposition of the studied block. As the deposition tool traverses during the deposition of each layer, as seen in the bottom graph, the recorded point’s temperature changes. For instance, this temperature ranges from 95 to 177 degrees Celsius while depositing the first layer, whereas it ranges from 168 to 185 degrees Celsius when putting down the 30th layer. The maximum and minimum temperatures measured during the deposition of each layer are listed in Table 2 (also shown in Figure 9). During the deposition process, the deformation layer temperature is expected to follow the fluctuation pattern shown in Figure 9. However, the actual values must be significantly higher than the substrate temperatures shown in Figure 9.

Figure 5 presents the microhardness measurements taken from the as-deposited part’s cross-section along the three vertical lines (center, left, and right lines—see Figure 4) running in the building direction. The microhardness values on the part’s cross-section decrease in a nonlinear fashion along the building direction from top to bottom. Figure 8 and Figure 9 present an increase in the mean measured temperature of the thermocouple reading point as the part deposition proceeds. Any location in the first layer may be assumed to have a comparable temperature history, and the same can be said for any other point in the component. The thermal history of the various locations explains the reduction in the hardness through the height of the as-deposited piece. Compared to a point located in the above layer (in building direction), a point in the first bottom layer undergoes more heating and re-heating cycles and stays at a higher temperature for a longer amount of time. This explains why the points at various positions along the building direction have varied hardness values. Additionally, when heat escapes from the part through the side walls, regions in the component’s center maintain their higher temperature for a longer period of time. The measured hardness of the points along the center line is, thus, less than the measured hardness of the points along the left and right lines (see Figure 5).

### 4.3. Change of XRD Pattern after the AFSD Process

**Figure 10:**
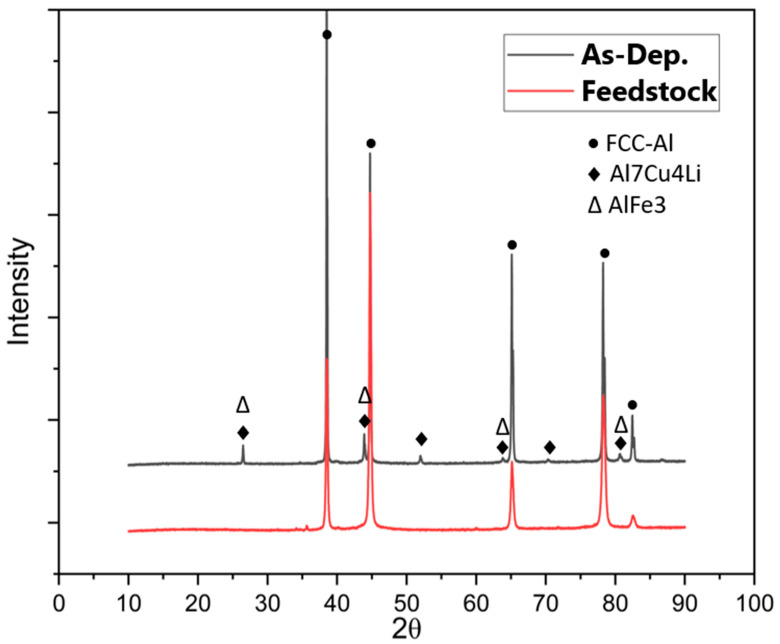
XRD patterns of the Al2050 feedstock used for AFSD processing and the as-deposited Al2050 part.

Figure 10 shows the X-ray diffraction (XRD) patterns of the used Al2050-T84 feedstock against the as-deposited Al2050 part at room temperature. Consistent with the Thermo-Calc prediction, the main peaks of the samples are the FCC-Al phase. However, after the AFSD process, the number of secondary phases increases significantly. In the feedstock matrix, only the FCC-Al can be observed. In the deposited Al2050 matrix, some secondary phase peaks are observed. It means that the alloying elements originally solidly dissolves in the FCC-Al matrix precipitate out and increase sizes to a level detectable by XRD. The formation of detectable new phases is believed to be the primary cause for the reduced microhardness test results of the AFSD components in contrast to those of the feedstock material, as shown by the hardness testing findings reported in Section 3. Based on Figure 7, the actual temperature values in the deformation layer are believed to be above 400 degrees Celsius.

### 4.4. Change of Composition Distribution of Al2050 after the AFSD Process

**Figure 11:**
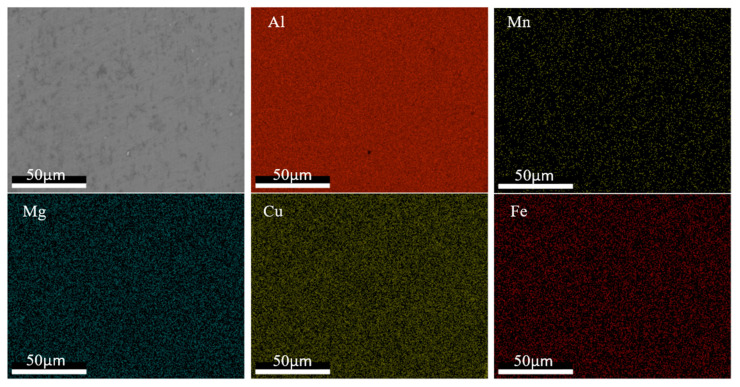
EDS mapping results showing the distribution of elements in the samples of the Al2050 feedstock material.

**Figure 12:**
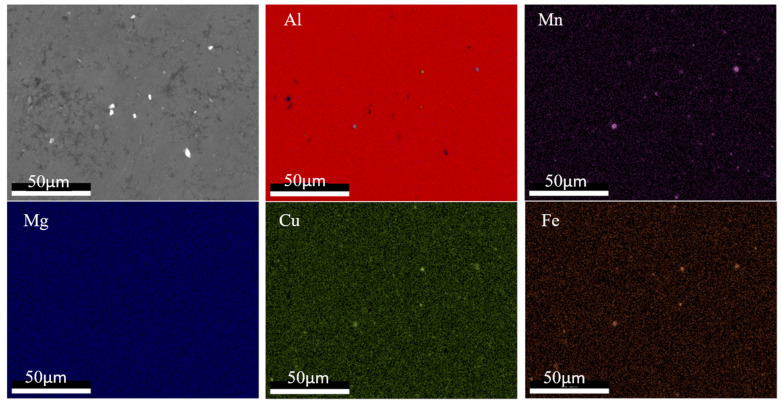
EDS mapping results showing the distribution of elements in the samples of the as-deposited Al2050 part.

The compositional distributions in the feedstock and the as-deposited (as-built) parts were investigated in this study. The EDS mapping was taken to elucidate the alloying element distribution. Figure 11 and Figure 12 show the EDS mapping for the Al2050 feedstock and as-deposited Al2050 sample, respectively. Based on the measurements, Al is observed as the matrix element. Mn, Cu, Mg, and Fe are obvious as the primary alloying element in the matrix. The difference between the feedstock and the as-deposited Al2050 is the shape and size of the alloying element enrichment region. In the feedstock matrix, the alloying element distributes uniformly, while in the as-deposited Al2050 matrix, three elements (Mn, Cu, and Fe) form detectable particles.

Based on XRD and EDS mapping, in the feedstock, the alloying elements are dispersed in the matrix FCC-Al. However, after the AFSD process, large-sized precipitates are formed, which reduce the hardness values.

During the AFSD process, the application of heat, generated by the friction between the rotating tool and the substrate, in conjunction with plastic deformation induced by the movement of the tool, creates conditions that can lead to dynamic recrystallization of the deposited material. The high strain rates and temperatures achieved at the stir zone promote this phenomenon [37,38]. Dynamic recrystallization results in a reduction in the average grain size, as well as an increase in the number of grains within the material. Zeng et al. studied the microstructure of Al6061 parts processed by AFSD [18]. For the as-deposited parts, the examination revealed equiaxed grains with a significantly reduced grain size, in comparison to the grain size of the feedstock material. Additionally, it was reported that there was no significant variance in the grain size along the direction of the build. As a result, it can be inferred that the variation in microhardness values along the direction of the build cannot be attributed to variations in grain size.

## 5. Conclusions

As the AFSD parts are subjected to a complex re-stirring and re-heating procedure during deposition, microstructural inhomogeneity inside the AFSD parts is examined by microhardness testing in this study. The Vickers microhardness test is carried out on both the feedstock rod and on the as-deposited AFSD Al2050 block. The measured microhardness values on the part’s cross-section decreases along the building direction from top to bottom. Although the microhardness for the top layer of the as-deposited Al2050 is the highest in the AFSD block, the value of 118.16 ± 2.90 HV is significantly smaller than the one for the feedstock (192.32 ± 6.52 HV). The temperature history experienced by the AFSD block during manufacturing, as well as the microstructures of the AFSD component and the feedstock, are investigated to elucidate the mechanism for hardness variation in the AFSD part. The measured temperature history proofs that for a location inside the component, the temperature fluctuates as the tool moves during the deposition. The component consequently undergoes multiple heating cycles, which affects the microstructure and, as a result, the mechanical properties of the AFSD parts. Using Thermo-Calc, the phases as a function of temperature are presented. Based on the XRD patterns for the feedstock material and the as-deposited part, secondary phases are detected in the deposited parts. Based on the EDS mapping, in the feedstock matrix, the alloying elements are distributed uniformly, while in the as-deposited Al2050 matrix, three elements (Mn, Cu, Fe) form detectable particles. The variation in the location specific temperature history is believed to be the primary cause for the variation in hardness values inside the AFSD block and for the reduced microhardness in contrast to those of the feedstock material.

## Figures and Tables

**Table 1:** Physical properties of wrought (0.5 in-1.5 in thick.) plate Al2050-T84 alloy (specification: AMS 4413) [27].

E, Modulus of Elasticity	E_c_, Modulus of Elasticity in Compression	G, Modulus of Rigidity	Poisson’s Ratio	Density
75 GPa (10.9 × 10^3^ ksi)	78 GPa (11.3 × 10^3^ ksi)	28.3 GPa (4.1 × 10^3^ ksi)	0.33	2713 kg/m^3^ (0.098 lb/in^3^)

**Table 2:** Max. and min. temperature in substrate during the deposition of each layer.

Layer No.	1	2	3	4	5	6	7	8	9	10	11	12	13	14	15
Min	95	83	145	122	165	141	177	151	186	161	185	163	188	167	178
Max	177	140	213	157	210	171	213	177	209	187	202	182	197	184	185
**Layer No.**	**16**	**17**	**18**	**19**	**20**	**21**	**22**	**23**	**24**	**25**	**26**	**27**	**28**	**29**	**30**
Min	160	186	166	183	164	186	167	193	172	198	176	187	171	180	168
Max	177	193	183	187	180	194	185	199	190	204	196	195	188	187	185

## Data Availability

The data presented in this study are available on request from the corresponding author.
